# Depletion of *Tcf3* and *Lef1* maintains mouse embryonic stem cell self-renewal

**DOI:** 10.1242/bio.022426

**Published:** 2017-03-13

**Authors:** Shoudong Ye, Tao Zhang, Chang Tong, Xingliang Zhou, Kan He, Qian Ban, Dahai Liu, Qi-Long Ying

**Affiliations:** 1 Center for Stem Cell and Translational Medicine, School of Life Sciences, Anhui University, Hefei 230601, People's Republic of China; 2 Eli and Edythe Broad Center for Regenerative Medicine and Stem Cell Research at USC, Department of Stem Cell Biology and Regenerative Medicine, Keck School of Medicine, University of Southern California, Los Angeles, CA 90033, USA

**Keywords:** Differentiation, Embryonic stem cells, LEF1, Self-renewal, TCF3

## Abstract

Mouse and rat embryonic stem cell (ESC) self-renewal can be maintained by dual inhibition of glycogen synthase kinase 3 (GSK3) and mitogen-activated protein kinase kinase (MEK). Inhibition of GSK3 promotes ESC self-renewal by abrogating T-cell factor 3 (TCF3)-mediated repression of the pluripotency network. How inhibition of MEK mediates ESC self-renewal, however, remains largely unknown. Here, we show that inhibition of MEK can significantly suppress lymphoid enhancer factor 1 (LEF1) expression in mouse ESCs. Knockdown or knockout of *Lef1* partially mimics the self-renewal-promoting effect of MEK inhibitors. Moreover, depletion of both *Tcf3* and *Lef1* enables maintenance of undifferentiated mouse ESCs without exogenous factors, cytokines or inhibitors. Transcriptome resequencing analysis reveals that LEF1 is closely associated with endoderm specification in ESCs. Thus, our study adds support to the notion that the key to maintaining the ESC ground state is to shield ESCs from differentiative cues.

## Abstract (precis)

**Summary:** Depletion of Lef1 and Tcf3 shows that ESCs could be shielded from differentiative cues to maintain the ESC ground state.

## INTRODUCTION

Mouse embryonic stem cells (mESCs) are derived from pre-implantation blastocysts and can be propagated extensively in culture while retaining the capacity to differentiate into all different cell types of the body (Evans and Kaufman, 1981; Huang et al., 2015; Martin, 1981). The maintenance of mESCs in an undifferentiated state can be achieved through activation of STAT3 by LIF (Niwa et al., 1998). We previously found that two small-molecule inhibitors (2i), CHIR99021 (CHIR) and PD0325901 (PD03), can also efficiently maintain mESC self-renewal independent of LIF/STAT3 signaling (Ying et al., 2008). CHIR stabilizes β-catenin through inhibition of GSK3. Stabilized β-catenin then abrogates the repressive action of TCF3 on the core pluripotency network function, and exerts its self-renewal effect in ESCs when the MEK pathway is suppressed simultaneously by PD03 (Wray et al., 2011; Yi et al., 2011; Ying et al., 2008). How inhibition of MEK by PD03 mediates ESC self-renewal, however, is still not fully understood.

In mESCs and early stage mouse embryos, TCF3 acts as a pro-differentiation factor by transcriptionally repressing the expression of pluripotency genes such as *Esrrb, Nanog, Tfcp2l1* and *Klf2* (Cole et al., 2008; Martello et al., 2012; Pereira et al., 2006; Qiu et al., 2015; Yi et al., 2008). Stabilization of β-catenin by CHIR alleviates the repressive effect of TCF3, and this has been hypothesized to be the key mechanism by which β-catenin promotes mESC self-renewal (Wray et al., 2011; Yi et al., 2011). Activation of β-catenin can also induce the expression of differentiation genes and the induction of these genes in ESCs depends on the interaction of β-catenin with LEF1 and TCF1, two of the four LEF1/TCF family members (Chatterjee et al., 2015; Chen et al., 2013). In this study, we found that the self-renewal-promoting effect of PD03 in mESCs is partially attributable to the suppression of *Lef1* expression and that depletion of *Tcf3* and *Lef1* can partially mimic the effect of 2i in maintaining ESC self-renewal.

## RESULTS AND DISSUSION

### CHIR down-regulates TCF3 in mESCs

**Fig. 1.**
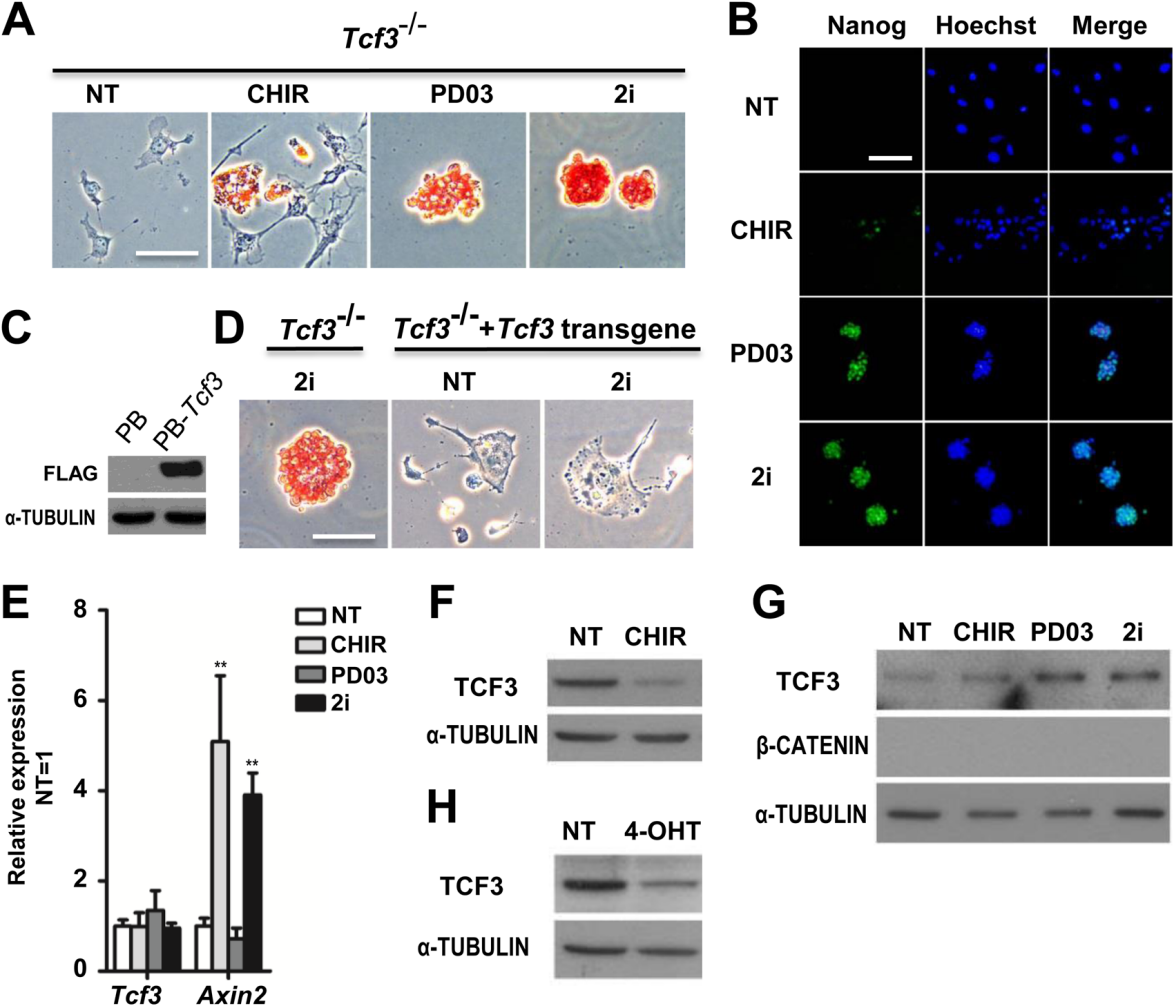
**CHIR promotes mESC self-renewal via down-regulation of TCF3 protein in a β-catenin-dependent manner.** (A,B) Alkaline phosphatase (AP) staining and immunofluorescence images of *Tcf3*^−/−^ mESCs cultured in N2B27 supplemented with the indicated small molecules for two passages. Hoechst, Hoechst 33342. (C) Western blot analysis of the expression of FLAG in *Tcf3*^−/−^ mESCs overexpressing FLAG-tagged *Tcf3* transgene. α-tubulin is a loading control. PB, PiggyBac. (D) AP staining of *Tcf3*^−/−^ mESCs and *Tcf3*^−/−^ mESCs overexpressing *Tcf3* transgene. Cells were cultured in N2B27 medium with or without 2i for two passages. (E,F) qRT-PCR (E) and western blot (F) analysis of *Tcf3* and *Axin2* expression in 46C ESCs cultured under the indicated conditions for 12 h. (G,H) Western blot analysis of TCF3 expression in *Ctnnb1* (β-catenin)^−/−^ ESCs (G) and *Ctnnb1*^−/−^ ESCs overexpressing *Ctnnb1-ERT2* (H). Cells were deprived of 2i/LIF overnight and then treated with the indicated compounds for 12 h in N2B27 medium. NT, no treatment; 4-OHT, 4-hydroxytamoxifen; ERT2, a mutant estrogen ligand-binding domain. Scale bars: 100 μm. Data represent mean±s.d. of three biological replicates. **P<0.01 vs NT.

*Tcf3^−/−^* mESC self-renewal could be maintained by PD03 alone (Fig. 1A,B), an outcome consistent with previous observations (Wray et al., 2011). Conversely, overexpression of TCF3 renders ESCs unable to self-renew in the 2i condition (Fig. 1C,D). These results confirm the strong connection between the self-renewal-promoting effect of CHIR and abrogation of the repressive action of TCF3 on the core pluripotency network (Wray et al., 2011). To investigate whether CHIR can directly regulate the expression of *Tcf3*, we treated mESCs with CHIR for 12 h and examined the expression of *Tcf3* by quantitative RT-PCR (qRT-PCR) and western blot analysis. While CHIR treatment significantly induced the expression of *Axin2*, a direct target of the Wnt/β-catenin pathway, this treatment elicited no effect on the expression level of *Tcf3* mRNA (Fig. 1E). The amount of TCF3 protein, however, was dramatically reduced by CHIR treatment (Fig. 1F), consistent with previous findings (Atlasi et al., 2013; Shy et al., 2013). CHIR treatment did not down-regulate TCF3 in *Ctnnb1^−/−^* mESCs (Fig. 1G); nuclear translocation of β-catenin led to decreased levels of TCF3 (Fig. 1H). These results confirm that the abrogation of TCF3's repressor function by CHIR might be achieved by degradation of TCF3.

### PD03 and LIF suppress the expression of *Lef1* in mESCs

**Fig. 2.**
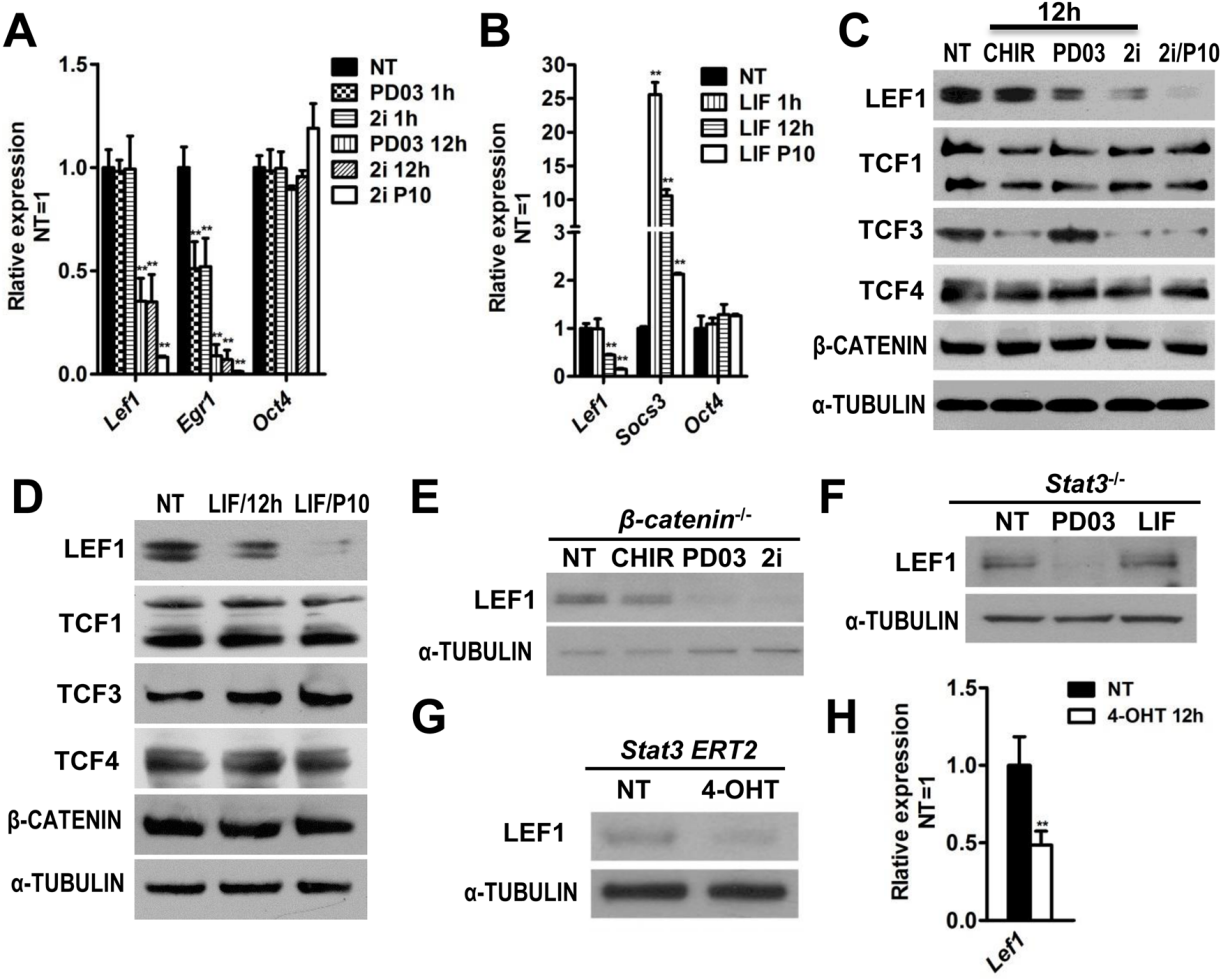
**Treatment with PD03 or LIF down-regulates *Lef1* expression in mESCs.** (A) qRT-PCR analysis of *Lef1*, *Egr1* and *Oct4* expression in 46C mESCs treated with PD03 or 2i for 1 h or 12 h in N2B27 medium after mESCs were deprived of 2i/LIF overnight. *Egr1* is a target gene of the MEK/ERK signaling pathway. *Oct4* is a stem cell pluripotency marker. 2i P10 and LIF P10 are the pluripotent baseline culture medium N2B27/2i and Serum/LIF, and mESCs were cultured in these conditions for more than 10 passages (P10). (B) qRT-PCR analysis of *Lef1* and *Socs3* expression in 46C mESCs treated with LIF for 1 h or 12 h in serum after mESCs were deprived of LIF overnight. *Socs3* is a target gene of the LIF/STAT3 signaling pathway. (C) Western blot analysis of 46C mESCs treated with the indicated small molecules for 12 h. (D) Western blot analysis of 46C mESCs treated with or without LIF for 12 h. (E-G) Western blot analysis of LEF1 expression in *Ctnnb1* (β-catenin)^−/−^ mESCs (E), *Stat3*^−/−^ mESCs (F) and *Stat3*^−/−^ mESCs overexpressing *Stat3-ERT2* transgene (G). Cells were treated with the indicated compounds for 12 h after deprivation of exogenous factors overnight. (H) qRT-PCR was used to detect the expression level of *Lef1* in *Stat3-ERT2*-overexpressing mESCs. Data represent mean±s.d. of three biological replicates. **P<0.01 versus NT. NT, no treatment.

CHIR functions in both self-renewal and differentiation in mESCs, and addition of PD03 or LIF can suppress the differentiation-inducing effect of CHIR to enable self-renewal under feeder- and serum-free conditions (Wray et al., 2011; Ying et al., 2008). It has been suggested that induction of differentiation genes by CHIR in rat and human ESCs is largely attributed to the abundance of LEF1 (Chen et al., 2013; Estarás et al., 2015). This prompted us to examine whether PD03 and LIF inhibit ESC differentiation induced by CHIR through down-regulation of LEF1. The expression of *Lef1* mRNA did not change significantly after stimulation with PD03 or LIF for 1 h. However, treatment with PD03 or LIF for 12 h substantially down-regulated the expression levels of both LEF1 protein and *Lef1* mRNA (Fig. 2A,B), and the transcript and protein levels of *Lef1* is significantly lower in the steady-state mESCs (treated with 2i or LIF for more than ten passages) than in mESCs treated with 2i or LIF for 12 h after overnight starvation, suggesting that LEF1 is not a direct target of PD03 and LIF. The expression levels of the other three TCF family members were not significantly altered by PD03 or LIF treatment (Fig. 2C,D).

Down-regulation of LEF1 by PD03 is likely independent of Wnt/β-catenin and LIF/STAT3 signaling, because PD03 treatment also significantly decreased the amount of LEF1 protein in *Ctnnb1^−/−^* and *STAT3^−/−^* mESCs (Fig. 2E,F). LIF-induced down-regulation of LEF1, however, is likely mediated by STAT3, because the LEF1 protein level in *STAT3^−/−^* mESCs did not change after LIF treatment. (Fig. 2F). To further confirm this result, we introduced a *STAT3-ERT2* transgene into *STAT3^−/−^* mESCs. Administration of 4-OHT to *STAT3-ERT2*-expressing cells results in the translocation of STAT3-ERT2 into the nucleus and the subsequent activation of STAT3 targets (Matsuda et al., 1999; Ye et al., 2016). As expected, 4-OHT treatment significantly down-regulated LEF1 expression in mESCs overexpressing *STAT3-ERT2* (Fig. 2G,H). Together, these data suggest that PD03 and LIF can down-regulate LEF1 expression in mESCs through independent mechanisms.

### Knockdown of *Lef1* partially mimics the differentiation-inhibiting effect of PD03

**Fig. 3.**
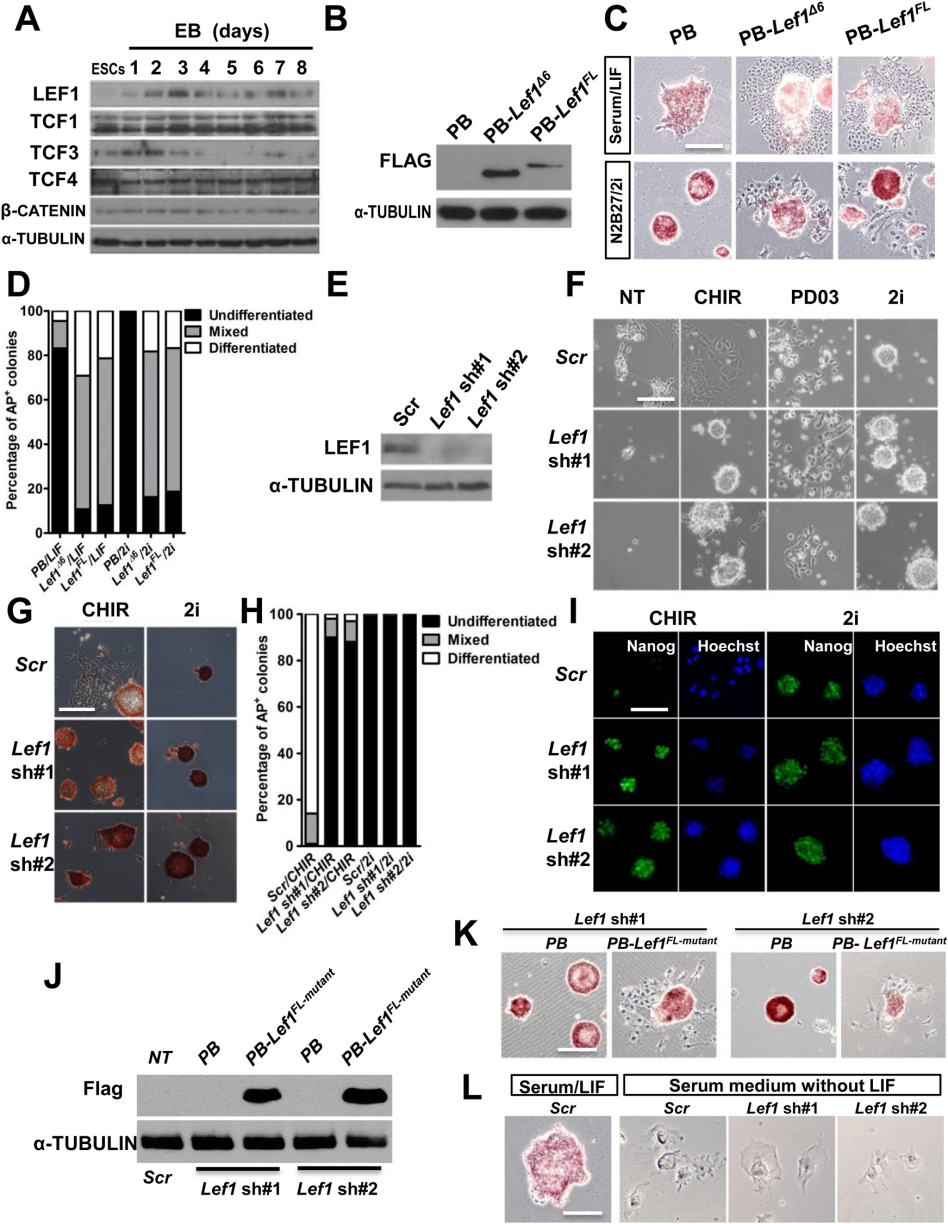
**Knockdown of *Lef1* partially mimics the self-renewal-promoting effect of PD03, but not LIF.** (A) Western blot analysis of TCF factors in 46C mESCs and 46C mESCs-derived embryoid bodies (EBs). (B) Western blot analysis of FLAG-tagged LEF1 in mESCs cultured in LIF/2i. (C,D) AP staining images of 46C mESCs overexpressing LEF1 and quantification of AP-positive colonies shown in Fig. 3C. Cells were cultured in the indicated conditions for two passages. (E) Western blot analysis of LEF1 expression in *Lef1* shRNA knockdown 46C mESCs. (F,G) Phase-contrast and AP staining of *Lef1*-knockdown and scramble control (Scr) mESCs cultured in the indicated conditions for five passages. (H) Quantification of AP-positive colonies shown in Fig. 3G: 500 colonies were counted under a microscope and classified as differentiated, undifferentiated or mixed. (I) Immunofluorescence staining of OCT4 in Scr control and *Lef1*-knockdown mESCs cultured in N2B27/CHIR or N2B27/2i for five passages (J) Western blot analysis of FLAG-tagged *Lef1^FL-mutant^* (PB-*Lef1^FL-mutant^*) in *Lef1*-knockdown mESCs. (K) AP staining images of *Lef1* sh#1 and *Lef1* sh#2 mESCs transfected with *Lef1^FL-mutant^* transgene and cultured under 2i/LIF condition for two passages. (L) AP staining of 46C mESCs, cultured in serum/LIF condition for 10 days, Scr control and *Lef1-*knockdown mESCs, cultured in serum for 10 days without LIF. Scale bars: 100 μm.

Next, we investigated whether suppression of LEF1 expression can mimic the effect of PD03 or LIF in the maintenance of ESC self-renewal. The expression of LEF1 was low in undifferentiated mESCs maintained in 2i/LIF but increased significantly in the first 24 h after mESCs were transferred to differentiation medium, while the levels of TCF1 and TCF4 were unchanged and TCF3 level decreased from day 3 onward (Fig. 3A), suggesting that up-regulation of LEF1 expression may be associated with the initiation of mESC differentiation. LEF1 has two isoforms, the full-length LEF1 (LEF1^FL^) and the alternative LEF1 transcript lacking exon 6 (LEF1^Δ6^) (He et al., 2008). To examine the function of LEF1 in mESCs, we generated 46C mESC lines overexpressing either flag-tagged LEF1^FL^ or LEF1^Δ6^ (Fig. 3B). ESCs transfected with empty vector remained undifferentiated, whereas the edge of many ESC colonies overexpressing *Lef1* was flat and lost alkaline phosphatase (AP) activity after two passages in N2B27/2i or serum/LIF condition (Fig. 3C,D), suggesting that elevated *Lef1* expression induces ESC differentiation. Next, we designed short hairpin RNAs (shRNAs) to knock down *Lef1* expression in 46C mESCs (Fig. 3E). ESCs stably expressing scramble shRNA remained undifferentiated in 2i, but differentiated in CHIR or PD as expected. In contrast, mESCs transfected with *Lef1* shRNA (*sh#1 or sh#2*) could be maintained in CHIR alone (Fig. 3F). sh*Lef1* mESCs cultured in CHIR could be continually passaged by single-cell dissociation while retaining expression of pluripotency markers (Fig. 3G-I). Similar results were obtained with another mESC line (Fig. S1A-C). Moreover, overexpressing *Lef1^FL^* harboring synonymous mutations at the sites targeted by our *Lef1* shRNA was able to rescue the phenotype induced by *Lef1* shRNA constructs, demonstrating the specificity of *Lef1* shRNA effect on self-renewal (Fig. 3J,K). These results suggest that suppression of *Lef1* expression can replace the requirement of PD03 for mESC self-renewal under the 2i condition. Although 46C ESCs can be routinely maintained in serum medium when supplemented with LIF and LIF treatment significantly down-regulates Lef1 (Fig. 2B,D), knockdown of *Lef1* expression is not sufficient for the maintenance of ESCs cultured in serum without LIF (Fig. 3L). This is unsurprising, given that multiple downstream targets of LIF have been identified to have a self-renewal-promoting effect (Martello et al., 2013; Ye et al., 2013). It is likely that down-regulation of *Lef1* together with activation of these LIF targets is required to recapitulate the self-renewal effect of LIF.

### Depletion of *Tcf3* and *Lef1* maintains mESC self-renewal

**Fig. 4.**
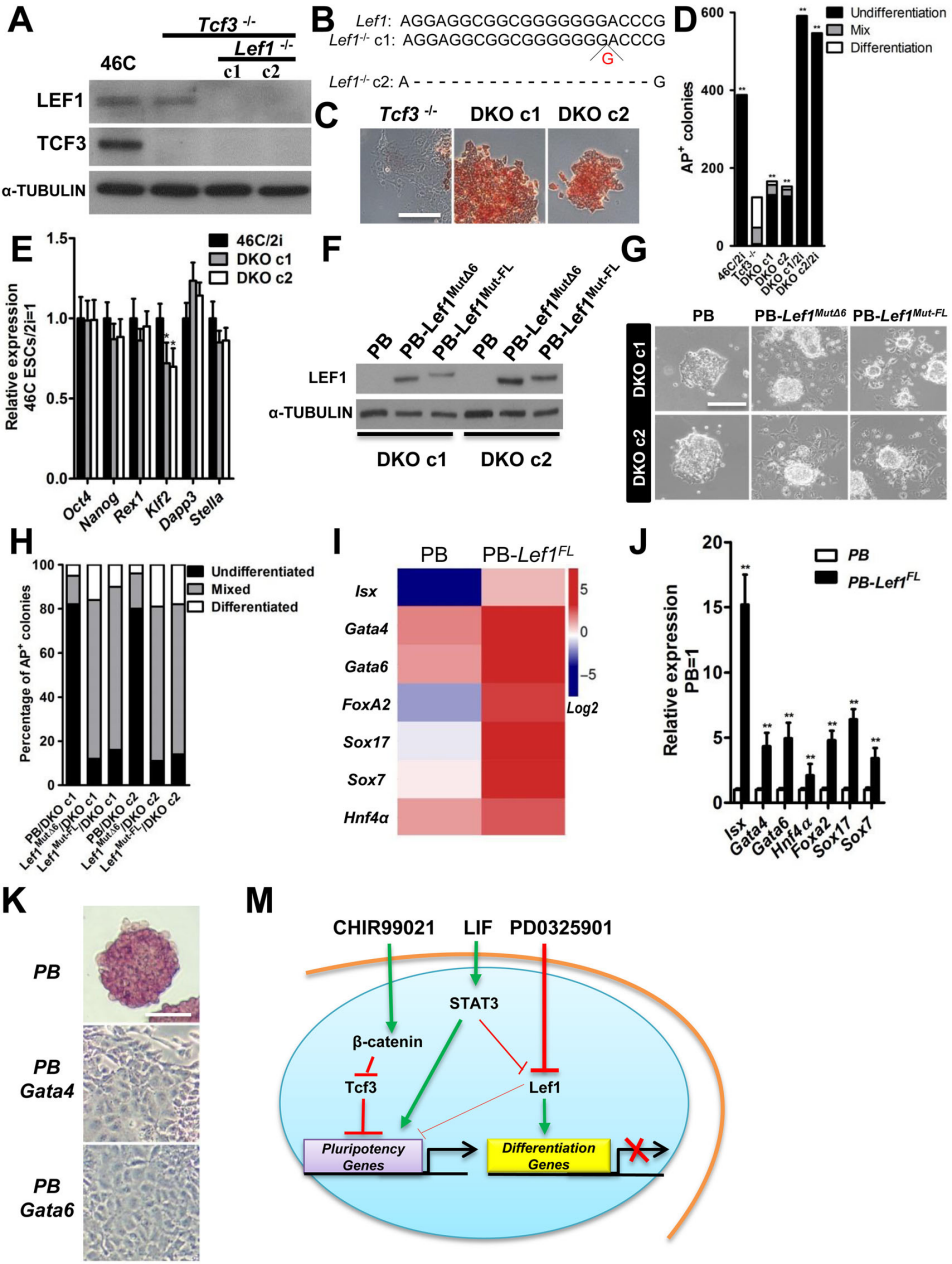
**Depletion of *Tcf3* and *Lef1* promotes mESC self-renewal.** (A) Western blot analysis of LEF1 and TCF3 expression in the indicated cells cultured in serum/LIF condition. c1, clone1; c2, clone 2. (B) Disruption of LEF1 by TALEN was verified by sequencing the genomic DNA. (C) Approximately 2000 cells were cultured in N2B27 only for 7 days and AP staining was performed. (D) Quantiﬁcation of AP-positive, AP-negative, and mixed mESC colonies shown in Fig. 4C. **P<0.01 vs *Tcf3^−/−^*. (E) qRT-PCR analysis of indicated pluripotency markers in 46C mESCs, cultured in 2i condition for 7 days, and DKO cells, cultured in N2B27 for 7 days. Data represent mean±s.d. of three biological replicates. **P<0.01 vs 46C/2i. (F) Western blot analysis of LEF1 expression in *Lef1* and *Tcf3* DKO cells transfected with *Lef1* synonymous mutants. (G) Phase-contrast images of the indicated mESCs cultured in N2B27 for five passages. (H) Quantification of AP-positive colonies. (I) Heat map showing the indicated gene expression pattern in PB and PB-*Lef1^FL^* mESC lines cultured in 2i/LIF for two passages. (J) Confirmation of the indicated gene expression in mESCs by qRT-PCR. Data represent mean±s.d. of three biological replicates. **P<0.01 vs PB. (K) AP staining images of PB, PB-*Gata4* and PB-*Gata6* 46C mESCs cultured in N2B27/2i for three passages. (M) Model of mESC self-renewal mediated by CHIR, PD03 and LIF. TCF3 and LEF1 are the two key transcription factors responsible for inducing mESC differentiation. TCF3 does so by repressing the expression of pluripotent genes while LEF1 induces mESC differentiation through induction of differentiation genes and suppressing pluripotency genes. CHIR and PD03 promote mESC self-renewal partly through down-regulation of TCF3 and LEF1. Although LIF/STAT3 signaling is able to suppress *Lef1* expression, it maintains mESC pluripotency mainly via inducing pluripotency gene expression in the presence of serum. Scale bars: 100 µm.

Since suppression of TCF3 or LEF1 expression can partially mimic the effects of CHIR or PD03, respectively, in the maintenance of mESCs, we sought to determine whether depletion of both TCF3 and LEF1 would enable mESC self-renewal in the absence of 2i. We designed gene-targeting vectors to knock out the *Lef1* gene in *Tcf3^−/−^* mESCs through transcription activator-like effector nuclease (TALEN)-mediated DNA double-strand breaks (Fig. S2A,B). After gene transfection and selection, we picked and expanded 15 colonies in the presence of LIF and 2i. The disruption of both *Lef1* alleles was confirmed in two clones by western blot analysis and genomic DNA sequencing (Fig. 4A,B). When transferred to N2B27 medium and cultured in the absence of LIF and 2i, *Tcf3/Lef1* double knockout (DKO) mESCs retained a typical undifferentiated ESC morphology and stained positive for AP even after long-term culture, whereas *Tcf3^−/−^* mESCs differentiated after 2-3 passages (Fig. 4C,D). Moreover, the expression levels of pluripotency genes were similar between DKO ESCs cultured in N2B27 and wild-type mESCs, cultured in N2B27/2i (Fig. 4E). Nonetheless, addition of 2i could still further augment self-renewal of *Tcf3/Lef1* DKO mESCs, and 46C mESCs cultured in 2i cells formed more AP-positive colonies than *Tcf3/Lef1* DKO mESCs (Fig. 4D). This is expected as CHIR and PD03 have also been shown to promote mESC self-renewal by inducing the expression of various pluripotency genes (Martello et al., 2012; Qiu et al., 2015; Yeo et al., 2014). It would be of interest to know if CHIR and PD03 induce the expression of these genes through suppression of TCF3 and LEF1 expression.

To rule out the possibility that these DKO mESCs might have undergone transformation to acquire the ability to self-renew independent of LIF and 2i, we reintroduced LEF1 isoforms into DKO mESCs and performed a self-renewal assay. We generated LEF1^FL^ and LEF1^Δ6^ mutants (LEF1^Mut-FL^ and LEF1^Mut-Δ6^) containing synonymous mutations at the two sites bound by LEF1-targeting TALENs to render them invisible to this targeting without compromising their native LEF1 functionality (Fig. S2C). We established DKO mESCs overexpressing LEF1^Mut-FL^ and LEF1^Mut-Δ6^ under the LIF/2i condition (Fig. 4F). When transferred to the N2B27 only culture condition, DKO mESCs transfected with an empty vector remained undifferentiated, whereas DKO mESCs overexpressing LEF1^Mut-FL^ or LEF1^Mut-Δ6^ differentiated (Fig. 4G,H). To further confirm that depletion of both TCF3 and LEF1 enables mESC self-renewal in the absence of 2i, we used shRNAs to knock down *Tcf3* and *Lef1* expression in Rex1-GFP mESCs in which a GFP reporter was knocked into the *Rex1* loci (Fig. S3A) (Toyooka et al., 2008). *Tcf3/Lef1* DKO mESCs maintained in N2B27 remained positive for REX1-GFP, whereas ESCs transfected with scramble shRNA differentiated (Fig. S3B,C). Taken together, these results demonstrated that depletion of both TCF3 and LEF1 can mimic the effect of 2i in the maintenance of mESC self-renewal.

### *Lef1*-overexpressing mESCs show enhanced endodermal specification

Since *Lef1* expression has been associated with the induction of differentiation-related genes in mESCs by CHIR, we next investigated how forced expression of *Lef1* affects the global gene expression pattern in mESCs. We performed RNA-sequence to assess the gene expression pattern of mESCs expressing PiggyBac (PB) vector or PB-*Lef1^FL^* (GEO Number: GSE77330). Compared with PB mESCs, PB-*Lef1*^FL^ mESCs showed an upregulation of a panel of endodermal markers, such as *Isx*, *Sox17*, *Sox7*, *HNF4a*, *Gata4*, *Gata6* and *Foxa2* .This expression pattern was further confirmed by qRT-PCR (Fig. 4I,J). Some of these genes have been shown to be strongly associated with mESC differentiation (Capo-Chichi et al., 2010). As expected, when overexpressed, both *Gata4* and *Gata6* rapidly induced mESC differentiation under the 2i condition (Fig. 4K). These data suggest that increased expression of LEF1 might initiate mESC exit from naïve pluripotency via inducing endodermal gene expression. LEF1 is closely associated with many differentiation activities in ESCs and during mammalian development (Galceran et al., 2001; Merrill et al., 2001; van Genderen et al., 1994; Zhou et al., 1995), and also has been shown to strongly inhibit the reprogramming of somatic cells to induced pluripotent stem cells (Ho et al., 2013). Further studies are needed to understand the mechanism by which LEF1 induces ESC differentiation and inhibits reprogramming. Inhibition of Wnt signal has been shown to be associated with enhanced expression of pluripotency markers and reduced differentiation in ESCs (Chatterjee et al., 2015; Faunes et al., 2013). It would be of interest to know whether LEF1 induces ESC differentiation through suppression of pluripotent gene expression.

To conclude, our findings indicate that TCF3 and LEF1 are the two key factors responsible for initiating exit from the naïve pluripotent state in mESCs. TCF3 does so by repressing the expression of pluripotency genes while LEF1 drives ESC differentiation through induction of lineage specification gene expression and suppression of pluripotency gene expression (Fig. 4M). We speculate that the function of TCF3 and LEF1 as the important differentiation-initiating factors is likely conserved among ESCs derived from different species. How the expression of TCF3 and LEF1 in ESCs is regulated by various extrinsic factors, however, might be subtly different among different species, and this might underlie the differences in the requirements for the maintenance of authentic ESCs from different species.

## MATERIALS AND METHODS

### Cell culture

The 46C mESCs were routinely cultured on 0.1% gelatin-coated plates in GMEM (Sigma) supplemented with 10% fetal calf serum (HyClone), 1% MEM NEAA (Invitrogen), 2 mM GlutaMax (Invitrogen), 0.1 mM β-mercaptoethanol (Invitrogen), and 1000 U/ml LIF (Millipore). For serum-free culture, mESCs were maintained in N2B27 supplemented with 3 µM CHIR99021 and 1 µM PD0325901 (Sigma).

### Generation of *Lef1*-knockout mESCs

Golden Gate TALEN and TAL Effector Kit 2.0 were purchased from Addgene. The RVD repeat arrays were assembled exactly as described by our previous report (Tong et al., 2012). The targeting colonies were picked and verified by using LEF1 antibody (C-19 or N-17, Santa Cruz, 1:500) or sequencing genomic DNA.

### Plasmid construction

The coding regions of *Lef1* were inserted into the PiggyBac vector. For RNA interference, we used plko.1-TRC (Addgene) system. The targeted sequences are GCGACTTAGCCGACATCAAGT (*Lef1* sh#1), GCATCCCTCATCCAGCT ATTG (*Lef1* sh#2) and GAAGGAAAGTGCAGCCATTAA (*Tcf3*). For generating the *Lef1^FL-mutant^*, the following mutations corresponding to *Lef1* shRNA-targeted regions were introduced: GCGATTTGGCAGATATTAAAT and GCATACCGCA CCCTGCGATCG.

### EB formation

For the EB formation assay, 1×10^7^ mouse 46C ESCs were grown using low-attachment dishes in standard ESC serum medium without LIF or inhibitors. The aggregates were allowed to grow for 8 days and samples were collected every day for western blot analysis.

### Western blotting, immunofluorescence staining and qRT-PCR

Western blotting, immunofluorescence staining and qRT-PCR were performed as previously reported (Ye et al., 2013). The primary antibodies used were LEF1 (N-17, Santa Cruz; 1:500), LEF1 (2286S, Cell Signaling Technology; 1:1000), TCF3 (M-20, Santa Cruz; 1:1000), TCF4 (H-125, Santa Cruz; 1:1000), TCF1 (2206S, Cell Signal Technology; 1:500), β-catenin (610154, BD Bioscience; 1:2000), and α-tubulin (32-2500, Invitrogen; 1:5000). The primers used are listed in Table S1.

### Statistical analysis

All data are reported as mean±s.d. A Student's *t*-test was used to determine the significance of differences in comparisons. Values of *P*<0.05 were considered statistically significant.
